# Molecular Mechanisms of *Lobelia nummularia* Extract in Breast Cancer: Targeting EGFR/TP53 and PI3K-AKT-mTOR Signaling via ROS-Mediated Apoptosis

**DOI:** 10.3390/cimb47070546

**Published:** 2025-07-14

**Authors:** Fahu Yuan, Yu Qiao, Zhongqiang Chen, Huihuang He, Fuyan Wang, Jiangyuan Chen

**Affiliations:** 1 College of Life and Health Sciences, Wuhan University of Science and Technology, Wuhan 430065, China; yuanfh@wust.edu.cn; 2 School of Medicine, Jianghan University, Wuhan 430056, China; yu.qiao@tum.de (Y.Q.); zqchen@jhun.edu.cn (Z.C.); huihuanghe@hotmail.com (H.H.); wangfy@jhun.edu.cn (F.W.); 3 School of Medicine and Health, Technical University of Munich, 81675 Munich, Germany

**Keywords:** triple-negative breast cancer (TNBC), natural products, luteolin, apigenin, oxidative stress, network pharmacology, serum pharmacochemistry, 4T1 orthotopic model

## Abstract

*Lobelia nummularia* Lam. is a traditional medicinal herb of which the anticancer mechanisms remain largely unexplored. Here, we demonstrated that its ethanolic extract (LNE) exerts potent anti-breast cancer activity by inducing ROS-dependent mitochondrial apoptosis in MDA-MB-231 cells, a mechanism confirmed via rescue experiments with the antioxidant N-acetylcysteine (NAC). This pro-apoptotic program is driven by a dual mechanism: potent suppression of the pro-survival EGFR/PI3K/AKT signaling pathway and simultaneous activation of the TP53-mediated apoptotic cascade, culminating in the cleavage of executor caspase-3. Phytochemical analysis identified numerous flavonoids, and quantitative HPLC confirmed that key bioactive compounds, including luteolin and apigenin, are substantially present in the extract. These mechanisms translated to significant in vivo efficacy, where LNE administration suppressed primary tumor growth and lung metastasis in a 4T1 orthotopic model in BALB/c mice. Furthermore, a validated molecular docking protocol provided a plausible structural basis for these multi-target interactions. Collectively, this study provides a comprehensive, multi-layered validation of LNE’s therapeutic potential, establishing it as a mechanistically well-defined candidate for natural product-based anticancer drug discovery.

## 1. Introduction

*Lobelia nummularia* Lam. (syn. *Pratia begonifolia* (Wall.) Lindl., *Pratia nummularia*) is a perennial herb of the genus *Lobelia*, widely distributed across China and Southeast Asia [1]. It is both edible and medicinal, and is frequently used in Traditional Chinese Medicine (TCM). In TCM, *L. nummularia* is traditionally employed to clear heat, eliminate toxins, reduce swelling, and promote blood circulation. These properties have led to its use in managing inflammatory and gynecological disorders. Despite its long history of clinical use, systematic pharmacological research into its antitumor potential remains limited.

Despite its traditional medicinal use, the phytochemical profile of *L. nummularia* has not been extensively studied. Ho [2] reported the isolation of two rare alkaloids from the plant, while Matsuura et al. [3] identified four flavonoids—diosmin, linarin, apigenin 7-O-rutinoside, and luteolin 7-O-rutinoside—as well as polyacetylenes from its aerial parts using NMR spectroscopy. These findings support the presence of diverse bioactive compounds potentially linked to its pharmacological effects.

Breast cancer remains the most frequently diagnosed malignancy among women worldwide, characterized by high incidence, heterogeneity, and treatment resistance [4,5]. Although current therapies such as surgery, chemotherapy, hormone therapy, and targeted drugs have improved outcomes, their adverse effects and limitations necessitate novel complementary treatments. Natural products, especially those derived from traditional medicines, are a rich source of multi-target agents with favorable safety profiles. Given the complex pathogenesis of breast cancer, plant-based therapeutics that exert pleiotropic effects are of particular interest [6,7,8].

The PI3K-AKT-mTOR signaling axis is a central pathway promoting tumor cell survival, proliferation, and metabolism [9]. Aberrant activation of this pathway is implicated in triple-negative breast cancer (TNBC), a subtype lacking hormone receptors and HER2 expression. In parallel, EGFR, an upstream receptor tyrosine kinase, plays a crucial role in modulating downstream PI3K-AKT signaling and is frequently overexpressed in TNBC [10]. Meanwhile, TP53, a tumor suppressor activated under cellular stress, can trigger mitochondrial apoptosis when oncogenic signaling is dysregulated [11]. Intriguingly, EGFR destabilization has been reported to enhance TP53 activation, leading to mitochondrial outer membrane permeabilization, cytochrome c release, and caspase cascade activation [12].

Reactive oxygen species (ROS) are important signaling molecules involved in tumor development and treatment [13]. Moderate levels of ROS can promote tumor growth, while excessive accumulation leads to oxidative stress and mitochondrial damage, triggering apoptosis. Natural anticancer agents such as paclitaxel exert their effects by increasing intracellular ROS levels [14]. This process results in mitochondrial membrane potential (MMP) loss, cytochrome c release, and caspase cascade activation [15,16,17]. These findings underscore the promise of natural products as multi-target therapeutics against oxidative stress-associated cancers such as breast cancer.

In this study, we established a comprehensive workflow to systematically investigate the anticancer mechanism of *Lobelia nummularia* extract (LNE). This approach integrates phytochemical characterization, including quantitative analysis of key bioactive flavonoids, with network pharmacology, and validated molecular docking to build a strong mechanistic hypothesis. We then experimentally interrogated these predictions in vitro and in vivo. Crucially, we sought to establish a causal link between reactive oxygen species (ROS) and apoptosis through rescue experiments, and subsequently confirmed the modulation of the PI3K/AKT and EGFR/TP53 pathways down to the level of the final executor caspase-3.

## 2. Materials and Methods

### 2.1. Preparation of Ethanolic Extracts of Lobelia nummularia

**Figure 1:**
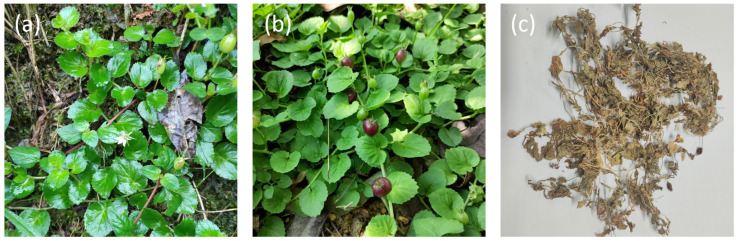
Representative images of *Lobelia nummularia* Lam. (**a**) Plants growing in the wild habitat showing green fruits and purple-white flowers; (**b**) field plants with partially ripened fruits, exhibiting a red-purple hue; (**c**) the dried whole-plant sample of *Lobelia nummularia* used in this study.

*Lobelia nummularia* plant was collected from Qiannan Prefecture, Guizhou Province, China (26.25° N, 107.52° E) (Figure 1). The voucher specimen is deposited in the Life Science Museum of Jianghan University. The preparation of the ethanol extract of *Lobelia nummularia* is briefly summarized as follows: dried *Lobelia nummularia* was crushed and passed through a 30-mesh sieve. The powder of *Lobelia nummularia* was taken and extracted by a reflux extraction with 80% ethanol three times for 60 min each. After filtering to remove the insoluble part, the extract was dried by rotary evaporation under reduced pressure using a rotary evaporator. Finally, the concentrated extract of *Lobelia nummularia* was obtained. The extract dried by vacuum freeze-drying was then sealed and stored at 4 °C until use.

### 2.2. Phytochemical Analysis

#### 2.2.1. Total Flavonoid Content (TFC)

The determination of the total flavonoid content (TFC) in LNE was based on the colorimetric method of NaNO_2_-Al(NO_3_)_3_-NaOH [18]. Using rutin as the standard substance, a standard curve was plotted, and the linear regression equation was obtained: Y = 10.332x + 0.0218 (correlation coefficient R^2^ = 0.999). The extract was diluted to the required concentration. After the same treatment, the absorbance value was measured at a wavelength of 510 nm, and the TFC content was calculated by substituting it into the linear regression equation, with the results expressed in terms of rutin equivalent (RE).

#### 2.2.2. Total Phenolic Content (TPC)

The Folin–Ciocalteu method [19] was used to determine the total phenolic content (TPC) of LNE. Using gallic acid as the standard substance, a standard curve was plotted, and the linear regression equation was obtained: Y = 0.0161x + 0.0182 (correlation coefficient R^2^ = 0.999). The extract was diluted to the required concentration. After the same treatment, the absorbance value was measured at a wavelength of 750 nm, and the TPC content was calculated by substituting it into the linear regression equation, with the results expressed in terms of gallic acid equivalent (GAE)

#### 2.2.3. Quantitative Analysis of Key Flavonoids by HPLC

The contents of five key flavonoids (ferulic acid, luteolin, latifolin, apigenin, and acacetin) in the LNE extract were determined using a high-performance liquid chromatography (HPLC) system (Essentia LC-16, Shimadzu, Kyoto, Japan) equipped with a UV detector. Chromatographic separation was performed on a Shim-pack VP-ODS C18 column (4.6 × 250 mm, 5 µm). An isocratic elution was employed, with the mobile phase consisting of acetonitrile and 0.05 M sodium dihydrogen phosphate buffer (adjusted to pH 2.5 with phosphoric acid) at a ratio of 30:70 (*v*/*v*). The analysis was conducted at a column temperature of 30 °C and a flow rate of 1.0 mL/min, with detection at a wavelength of 276 nm. Standard stock solutions of the five compounds were prepared and serially diluted to establish calibration curves. The content of each compound in the LNE extract was calculated from the linear regression equation of its corresponding standard curve.

### 2.3. Antioxidant Assays In Vitro

The freeze-dried powder of the LNE extract was accurately weighed and diluted with ethanol in gradients to prepare LNE test working solutions with mass concentrations of 0.2, 0.4, 0.6, 0.8, and 1.0 mg/mL. Compared with vitamin C at the same mass concentration gradient, the scavenging abilities of DPPH radicals, ABTS cation radicals, and hydroxyl radicals were determined. The specific determination operations refer to the methods reported by Meng [20] and Chen [21].

### 2.4. Anticancer Activity In Vitro

#### 2.4.1. Cell Culture and LNE Administration

The human breast cancer cell line MDA-MB-231 and the mouse breast cancer cell line 4T1 were both obtained from the national collection of authenticated cell cultures. The cells were cultured in a DMEM (high glucose) medium containing 10% fetal bovine serum. The cell culture environment was carried out under humid conditions at 37 °C and 5% CO_2_. The medium was replaced every 48 h, and the cells at 70% confluence were used for experimental treatment. For the experimental group, different concentrations of the LNE extract were applied to the MDA-MB-231 cells; for the negative control group, the same volume of DMSO was applied in all experiments.

#### 2.4.2. Cell Proliferation Assay

The CCK-8 kit (cat#C0037, Beyotime Biotechnology, Shanghai, China) was used to determine the cytotoxicity of LNE against human (MDA-MB-231) and murine (4T1) TNBC cells, as well as non-cancerous human breast epithelial cells (MCF-10A). The cells were seeded into 96-well plates at a density of 2 × 10^4^ cells per well and cultured for 24 h to ensure cell adhesion. The cells were treated with gradient concentrations of LNE (25, 50, 100, 200, and 400 μg/mL) for 24 or 48 h. Then, a 10% CCK-8 solution was added to each well of the 96-well plate, and the plate was incubated at 37 °C for 2 h. The absorbance at 450 nm was measured using a microplate reader (Multiskan SkyHigh, Thermofisher, Waltham, MA, USA).

#### 2.4.3. Wound Healing Assay to Determine the Migration Ability of MDA-MB-231

MDA-MB-231 cells were seeded in 6-well plates at a density of 5 × 10^5^ cells per well. When the cells covered the plate, a scratch was made with a 10 μL pipette tip. The cells were washed twice with sterile PBS to remove the scratched cells. LNE prepared with a serum-free medium was added to the LNE group. The scratch at 0 h was photographed, and the photographing points were recorded. After culturing in an incubator at 37 °C and 5% CO_2_ for 12 h and 24 h, photographs were taken and the scratch distance was measured at the same photographing points.

#### 2.4.4. Hoechst Staining to Determine Cell Apoptosis

MDA-MB-231 cells were seeded in 6-well plates and placed in an incubator at 37 °C and 5% CO_2_ overnight. The cells were induced and treated with LNE for 48 h. The nuclear fragmentation and apoptotic cell morphology of the cells were examined by Hoechst staining. Then, 4% paraformaldehyde was used to fix the treated cells for 30 min, and they were stained with Hoechst 33342 fluorescent dye (cat#C0003, Beyotime Biotechnology, China) at a final concentration of 1 μg/mL for 10 min. After staining, the cells were washed three times with sterile PBS and observed using a fluorescence microscope.

#### 2.4.5. Study on Intracellular ROS Generation

MDA-MB-231 cells were seeded in 6-well plates, and induced and treated with LNE for 48 h. Then, the medium was removed, the cells were washed with PBS, and then treated with 10 μM H_2_DCF-DA dye (cat#S0033S, Beyotime Biotechnology, China). The cells were incubated at room temperature for 30 min. Cell images were taken using a fluorescence microscope.

#### 2.4.6. JC-1 Staining to Detect Mitochondrial Membrane Potential

JC-1 staining was used to detect the loss of MMP (ΔΨm) in MDA-MB-231 cells. MDA-MB-231 cells were seeded in 6-well plates and then incubated at 37 °C. These samples were treated with LNE for 48 h. After 48 h, the medium was removed, the cells were washed with PBS, 1 mL of JC-1 staining solution (cat#M8650, Solarbio Science & Technology, Beijing, China) was added, and the cells were placed in an incubator at 37 °C for 20 min. After incubation, the supernatant was removed, and the cells were observed and photographed under a fluorescence microscope.

#### 2.4.7. NAC Rescue Experiment and Flow Cytometry Analysis

To investigate the role of ROS in LNE-induced apoptosis, a rescue experiment was performed using the ROS scavenger N-acetylcysteine (NAC) (cat#ST2524, Beyotime Biotechnology, Shanghai, China). MDA-MB-231 cells were seeded in 6-well plates and allowed to adhere overnight. The cells were then divided into four groups: (i) a control group (vehicle-treated), (ii) an LNE-only group (50 µg/mL), (iii) an NAC-only group (5 mM]), and (iv) a combination group (NAC + LNE). For the combination group, cells were pre-treated with NAC for 2 h before the addition of LNE. After 48 h of treatment with LNE, cells were harvested for subsequent analysis.

For apoptosis analysis, the harvested cells were washed twice with cold PBS and then stained using an Annexin V-FITC/Propidium Iodide (PI) Apoptosis Detection Kit (cat#AP101C, Multi Sciences Biotech, Hangzhou, China) according to the manufacturer’s protocol. Briefly, cells were resuspended in a 1× binding buffer and incubated with Annexin V-FITC and PI for 15 min at room temperature in the dark. Stained cells were immediately analyzed on a BD C6 PLUS flow cytometer.

For intracellular ROS measurement, harvested cells were incubated with 10 µM of 2′,7′-dichlorodihydrofluorescein diacetate (DCFH-DA) (cat#S0033S, Beyotime Biotechnology, China) in a serum-free medium for 30 min at 37 °C in the dark. After incubation, cells were washed twice with PBS to remove the excess probe. The fluorescence intensity of DCF, which is proportional to the intracellular ROS level, was immediately measured by flow cytometry.

### 2.5. Preparation of Serum Samples to Identify Chemically Absorbed Substances In Vivo

Eight female Kunming strain mice (8-week-old) were randomly divided into two groups: the LNE serum group and the control serum group. After a 1-week adaptation period, the mice were administered LNE or distilled water via gavage, respectively. The LNE was prepared to a final concentration of 600 mg/mL and administered at a volume of 15 mL/kg to achieve a final dose of 8.96 g/kg. The mice were dosed once a day for 7 consecutive days. On the last day, there was an interval of 1 h between the two administrations for the mice. One hour after the last administration, blood was collected from the abdominal aorta, and then the blood was stored at room temperature for 1 h. The samples were centrifuged at 1500× *g* for 20 min at 4 °C, and then the serum was collected for subsequent liquid chromatography–tandem mass spectrometry (LC-MS/MS) analysis. The ethical review involving experimental animals has been approved by the Ethics Review Committee of the Experimental Animal Center of Jianghan University (protocol number JHDXLL2024-125), and all animal studies were conducted in accordance with the guidelines of the Animal Care and Use Committee of Jianghan University.

Of the serum sample, 300 μL was taken, 30 μL of hydrochloric acid (2 mol/L) added, and it was then vortexed and mixed for 1 min, and allowed to stand at 4 °C for 15 min. After repeating the vortexing and standing four times, 1.2 mL of acetonitrile was added, and vortexed and mixed for 5 min. Then, it was centrifuged at a centrifugal force of 13,800× *g* for 5 min to obtain the supernatant. Then, 1350 μL of the supernatant was taken and dried by nitrogen blowing, 112.5 μL of 80% methanol (with an internal standard concentration of 10 μg/mL) was added for reconstitution, and it was vortexed and mixed for 5 min, and centrifuged at a centrifugal force of 13,800× *g* for 5 min to obtain the supernatant. Finally, 100 μL of the supernatant was transferred to a vial for LC-MS analysis.

### 2.6. HPLC-QTOF/MS Analysis

The HPLC-Q-TOF/MS combined system consists of a Dionex Ultimate 3000 HPLC system (Dionex, Thermo Scientific, Waltham, MA, USA), together with a SCIEX Triple TOF 5600 mass spectrometer (AB SCIEX, Concord, ON, Canada) equipped with Analyst v1.6 workstation software, PeakView v2.2 qualitative screening software, and LibraryView v1.4 database software.

Chromatographic separation was performed using an Accucorea Q C18 column (150 mm × 2.1 mm, 2.6 μm, Thermo Scientific, USA). The column temperature was maintained at 35 °C, the injection volume was 5 μL, and the flow rate was set at 0.3 mL/min. Mass spectrometry analysis was performed using an electrospray ionization source (ESI) in both positive and negative ion modes. When using the positive ion scanning (ESI+) mode, the mobile phase A was acetonitrile, and the mobile phase B was an aqueous solution of 0.1% formic acid; when using the negative ion scanning (ESI−) mode, the mobile phase A was acetonitrile, and the mobile phase B was ultrapure water. Binary gradient elution was adopted, and the elution program was set as follows: 0–2 min, 5% A; 2–20 min, 5% A-95% A; 20–25 min, 95% A; 25–26 min, 95% A-5% A; 26–30 min, 5% A. The spray voltage was 5.5 kV in the ESI+ mode and 4.5 kV in the ESI− mode. The ion source temperature was 550 °C. The declustering potential was 60 V, the collision energy was 35 V, the scanning mode was full-scan primary mass spectrometry with a mass acquisition range of 100–1000 Da, and full-scan secondary mass spectrometry with a mass acquisition range of 50–1000 Da.

### 2.7. Network Pharmacology Analysis

#### 2.7.1. Database Construction

According to the above serum pharmacochemistry experiment, 65 serum prototype components could be identified after the LNE extract, and these components were used to construct the basis of potential effective components for network pharmacology. The online database swisstargetprediction (http://swisstargetprediction.ch, accessed on 4 June 2025) was used to obtain the potential target genes of the former. Then, “breast cancer” was used as a keyword to obtain the disease-related gene sets from the Disgenet database (https://www.disgenet.org, accessed on 4 June 2025) and the Genecards database (https://www.genecards.org, accessed on 4 June 2025). We linked the breast cancer-related genes with the potential target genes of LNE to obtain the intersections between them. These intersections were defined as the potential targets of LNE for treating breast cancer.

#### 2.7.2. Construction of the Protein–Protein Interaction Network

Proteins rarely function in isolation, and their interactions play a crucial role in promoting various cellular processes. To gain an in-depth understanding of the potential interactions of the drug targets we identified, we constructed a protein–protein interaction (PPI) network. For this purpose, we utilized the STRING database (https://string-db.org/, accessed on 4 June 2025), focusing on Homo sapiens proteins and selecting high-confidence interactions with a score exceeding 0.9. Subsequently, we used Cytoscape software (www.cytoscape.org/, accessed on 4 June 2025; version 3.10.1) to visually display this network.

#### 2.7.3. GO and KEGG Pathway Enrichment Analysis

To comprehensively elucidate the importance of the identified key genes, we conducted functional enrichment analysis using Gene Ontology (GO) and Kyoto Encyclopedia of Genes and Genomes (KEGG) pathways. GO annotation and KEGG pathway enrichment were carried out. We applied a statistical significance threshold of *p* < 0.05, and used bubble plots and bar charts to visually represent the enriched GO terms and KEGG pathways. By integrating these analysis methods, we obtained valuable insights into the functional roles of the identified key genes and identified the key pathways that may be affected by the compounds under study.

#### 2.7.4. Molecular Docking

Through molecular docking analysis, the interactions between the main components of LNE and the core target genes were visually characterized. We downloaded the 2D sdf structure files of five compounds, namely acacetin, apigenin, luteolin, diosmetin, and latifolin, from PubChem, and used the LigPrep module in Schrödinger’s computational platform for processing and generated all their 3D chiral conformations.

The crystal structures of target proteins were obtained from the RCSB PDB database: EGFR (PDB ID: 8A27), TP53 (PDB ID: 6GGC Chain A), STAT3 (PDB ID: 6NJS) HSP90AA1 (PDB ID: 5J2X) and AKT1 (PDB ID: 4GV1). These structures were selected based on criteria including high resolution, human origin, and the presence of a co-crystallized ligand to define the binding pocket. The obtained protein crystals were processed using the Protein Preparation Wizard module of Schrödinger’s computational platform, including protein preprocessing, regenerating states of native ligand, H-bond assignment optimization, protein energy minimization, and removing waters. First, the SiteMap module in Schrödinger’s computational platform was used to predict the optimal binding site, and then the Receptor Grid Generation module in Schrödinger’s computational platform was used to set the most appropriate Enclosing box to perfectly enclose the predicted binding site, and on this basis, the active sites of the five proteins were obtained. The five processed ligand compounds were, respectively, docked with the active sites of the five proteins. The lower the score, the lower the binding free energy of the compound to the protein, and the higher the binding stability. To validate the docking protocol, the co-crystallized ligand of EGFR was extracted and re-docked into the protein’s binding site using the same parameters. The accuracy of the protocol was confirmed by a low root mean square deviation (RMSD) of 0.134 Å between the docked pose and the original crystallographic pose, which is well below the 2.0 Å threshold for a successful validation.

### 2.8. Verification of Animal Studies

Six-week-old female BALB/c mice purchased from Vital River Laboratories were conventionally raised in the SPF-grade barrier environment of the Animal Experiment Center of Jianghan University. They had free access to water and food at 24 ± 1 °C and a relative humidity of 55 ± 5%. The 4T1 breast cancer cells in the logarithmic growth phase were treated with 0.25% trypsin to form a cell suspension, centrifuged at a low speed at room temperature, the cells resuspended with pre-cooled PBS and counted, and the cell density adjusted to 2 × 10^5^ cells/mL. A sterile syringe was used to inoculate 50 μL of the above cell suspension into the fourth mammary fat pad on the left side of the subcutaneous region of each mouse. Three days after the 4T1 cell inoculation, the tumor formation in the mice was observed to confirm the successful transplantation of breast cancer cells. Qualified breast cancer model mice were randomly divided into 3 groups with 5 mice in each group: (1) the vehicle (normal saline) treatment control group (control), (2) the 100 mg/kg body weight (BW)/day LNE treatment group (LNE-L), and (3) the 300 mg/kg body weight (BW)/day LNE treatment group (LNE-H). The sample size of five mice per group was determined based on established practices in similar preclinical oncology studies and in accordance with the 3Rs principles for animal welfare, providing sufficient statistical power to detect significant treatment effects. LNE was freshly dissolved in normal saline every day and administered via gavage for 25 consecutive days at a dose of 100 or 300 mg/kg BW/day. The required dose of LNE was administered daily via oral gavage in a volume of 10 mL/kg body weight. On the 28th day after tumor inoculation, the mice were sacrificed by the CO_2_ method. After depilation of the tumor-bearing site of the mice, photos were taken, and then the mice were dissected to remove the tumors for weighing and photographing the tumor tissues. The tumor volume was calculated according to the following equation: (width) × (length)^2^/2. The lungs and liver of the mice were removed, and the number of tumor metastasis foci was recorded. Subsequently, part of the tumor tissues, liver tissues, and lung tissues were cut and fixed with 4% paraformaldehyde tissue fixing solution for subsequent H&E staining and ki67 immunohistochemical detection. The remaining tissue samples were rapidly frozen in liquid nitrogen and stored at −80 °C.

### 2.9. Western Blot

Equal amounts of tumor tissue protein (30 μg) were separated by SDS-PAGE using 10% or 12% polyacrylamide gels and transferred onto PVDF membranes (Millipore, Billerica, MA, USA). Membranes were blocked with 5% BSA in TBST (0.1% Tween-20 in TBS) for 1 h at room temperature and then incubated overnight at 4 °C with primary antibodies against the following targets: p-TP53 (Ser15) (cat#9284), p-EGFR (Tyr1068) (cat#3777), p-PI3K p85 (Tyr458) (cat#17366), p-AKT (Ser473) (cat#4060), p-mTOR (Ser2448) (cat#2976), Bax(cat#2772), Bcl-2 (cat#3498), Cyt C (cat#11940), cleaved caspase-9 (cat#9509), total TP53 (cat#2524), total EGFR (cat#54359), total AKT (cat#9272), cleaved caspase-3 (cat#9661), and β-actin (cat#4967) (all from Cell Signaling Technology, Beverly, CA, USA). After washing, membranes were incubated with HRP-conjugated secondary antibodies (CST) for 1 h at room temperature.

Protein bands were visualized using an enhanced chemiluminescence (ECL) substrate (cat#WP20005, Thermo Fisher Scientific, USA), and imaged with a chemiluminescence imaging system (Tanon 4600, Shanghai, China). Band intensities were quantified using ImageJ software (version 1.52a) and normalized to β-actin as the internal control. All experiments were repeated at least three times independently.

### 2.10. Statistical Analysis

The results obtained in the in vitro assays were expressed as the mean ± standard deviation. One-way analysis of variance test and Dunnett’s multiple comparison were used to compare the groups. For statistical analysis, GraphPad Prism (v9.0.0, La Jolla, CA, USA) was used.

## 3. Results

### 3.1. Total Phenolic and Total Flavonoid Contents of Ethanolic Extract of Lobelia nummularia

Bioactive compounds are usually extracted from plant materials using a single solvent or a mixture of solvents. Commonly used solvents include water, ethanol, and methanol, as they have been proven to be effective in extracting polyphenols from plants to obtain phenolic compounds with high solubility in these solvents. In this study, 80% ethanol was selected as the solvent for the maceration extraction process. An ethanolic extract was obtained from *Lobelia nummularia*. Subsequently, the polyphenol and flavonoid contents of the extract of *Lobelia nummularia* were evaluated. The TPC value of the extract of *Lobelia nummularia* was 130.25 ± 6.48 mg GAE/g Extract. Flavonoids are a class of phenolic compounds, which are a group of naturally occurring compounds present in plants and possess pharmacological effects. In our study, the ethanolic extract of *Lobelia nummularia* showed that the flavonoid content of the extracted compounds was 114.58 ± 5.09 mg RE/g Extract.

### 3.2. Compounds in the Ethanolic Extract of Lobelia nummularia Identified by HPLC-QTOF-MS

The total ion extraction chromatogram obtained by HPLC-QTOF-MS showed that there were abundant plant secondary metabolites in LNE. Through accurate mass number determination, retention time, ion fragmentation behavior, reference standards, and relevant literature, a total of 75 compounds were identified from LNE (Table 1). The molecular weights of these plant components were in the range of 150–900 Da, including flavonoids, phenylpropanoids, sesquiterpenoids, triterpenoids, and terpenoids, etc.

### 3.3. Quantitative Analysis of Key Bioactive Flavonoids in LNE

To establish a robust phytochemical basis for LNE’s activity, a validated HPLC method was developed to quantify the five key flavonoids previously identified as potential core components by network pharmacology. The representative HPLC chromatograms of a standard mixture and an LNE sample are shown in Supplementary Figure S1, demonstrating effective separation of the target compounds. The analytical method was validated for its reliability; as detailed in Table 2, the calibration curves for all five compounds (ferulic acid, luteolin, latifolin, apigenin, and acacetin) exhibited excellent linearity, with all correlation coefficients (R^2^) exceeding 0.999.

Using this method, the contents of the five flavonoids in the LNE extract were determined. As summarized in Table 3, all five compounds were confirmed to be substantially present in the extract. Notably, ferulic acid and apigenin were among the most abundant of the quantified compounds, with contents of 6.25 ± 0.34 mg/g and 5.66 ± 0.35 mg/g, respectively. These quantitative data provide a clear material basis for the LNE used in this study, and support the hypothesis that these specific flavonoids are key contributors to its overall bioactivity.

### 3.4. Antioxidant Activity of Ethanolic Extract of Lobelia nummularia

**Figure 2:**
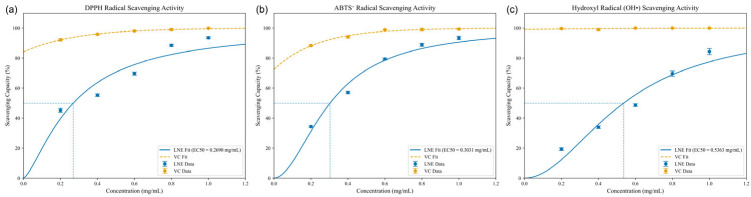
Antioxidant activities of LNE. (**a**) DPPH radical scavenging activity of LNE; (**b**) ABTS+ radical scavenging activity of LNE; (**c**) hydroxyl radical (OH^•^) scavenging activity of LNE. Data are presented as the mean ± SD from three independent experiments. LNE exhibited a dose-dependent antioxidant effect comparable to vitamin C, indicating its potent free radical scavenging ability.

Reactive oxygen species (ROS) are involved in many aspects of health and disease. There is a delicate balance between the oxidative stress and antioxidant mechanisms within cells to ensure that physiological functions are maintained. Any disruption of this balance may lead to pathological consequences. Antioxidant activity analysis may provide the direction and basis for further biological screening tests in the study. Here, we evaluated the antioxidant activity of LNE through experiments on the scavenging abilities of DPPH radicals, ABTS cation radicals, and hydroxyl radicals. The results are shown in Figure 1a–c. The antioxidant activity of LNE at all measured concentrations was dose-dependent, but to varying degrees. At the same concentration, the scavenging activities of DPPH, ABTS, and OH radicals of LNE were lower than those of the positive control (vitamin C). The IC50 values of the scavenging activities of DPPH, ABTS, and OH radicals of LNE were 0.27, 0.30, and 0.54 mg/mL, respectively (Figure 2). This indicates that LNE contains a large amount of antioxidants and has potential application potential.

### 3.5. LNE Induces Cytotoxicity in MDA-MB-231 Cells

**Figure 3:**
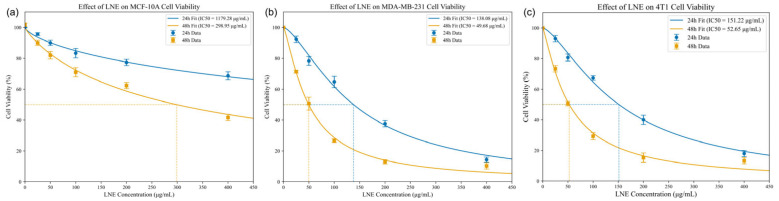
Effects of LNE on cell viability. (**a**) Cytotoxicity profile of LNE in non-cancerous human mammary epithelial MCF-10A cells. (**b**) Dose- and time-dependent cytotoxicity of LNE in human breast cancer MDA-MB-231 cells. (**c**) Dose- and time-dependent cytotoxicity of LNE in murine breast cancer 4T1 cells. All data were assessed by a CCK-8 assay.

The cytotoxic effects of LNE were evaluated on human (MDA-MB-231) and murine (4T1) TNBC cells, as well as on non-cancerous human breast epithelial cells (MCF-10A), using a CCK-8 assay. As shown in Figure 3, LNE inhibited the viability of all cell lines in a dose- and time-dependent manner. Notably, LNE was significantly more potent against the cancer cell lines. The 48 h IC50 values for MDA-MB-231 and 4T1 cells were 46.08 µg/mL and 52.65 µg/mL, respectively, indicating a comparably high sensitivity to LNE. In contrast, the non-cancerous MCF-10A cells were much less sensitive, with a 48 h IC50 of 298.95 µg/mL. This demonstrates LNE’s potent and selective cytotoxic activity against TNBC cells.

### 3.6. LNE Induces Apoptotic Morphology in MDA-MB-231 Cells

**Figure 4:**
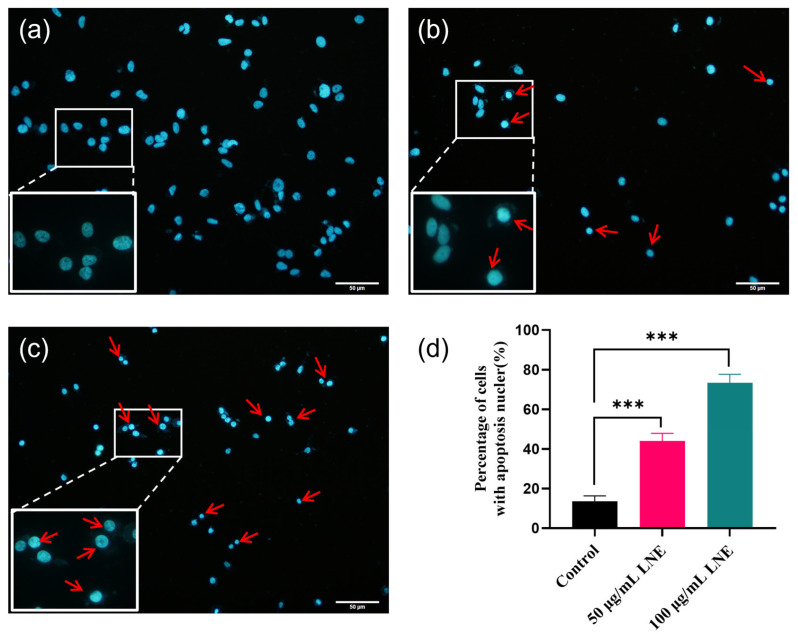
LNE-induced apoptosis in MDA-MB-231 cells visualized by Hoechst 33258 staining. (**a**) Control group showing normal nuclear morphology with uniform chromatin distribution; (**b**) cells treated with 50 μg/mL LNE for 48 h, exhibiting early apoptotic features such as chromatin condensation (indicated by arrows); (**c**) cells treated with 100 μg/mL LNE for 48 h, showing prominent nuclear fragmentation and apoptotic bodies (indicated by arrows); (**d**) quantification of apoptotic cells based on Hoechst-positive nuclei. Data are presented as the mean ± SD (*n* = 3). *** *p* < 0.01 vs. control group.

Upon the administration of LNE, fluorescence microscopy with Hoechst 33258 staining was used to investigate the apoptotic properties of MDA-MB-231 cells. After treatment with 50 and 100 μg/mL of LNE for 48 h and staining with Hoechst 33258, under the fluorescence microscope, the nuclei of the cells in the control group were normally blue. In contrast, treatment with LNE significantly promoted the apoptosis of MDA-MB-231 cells. The nuclei of the apoptotic cells were densely stained, or presented as fragmented and densely stained, and the color was somewhat whitish (Figure 4).

### 3.7. LNE Elevates Intracellular ROS and Disrupts Mitochondrial Membrane Potential

**Figure 5:**
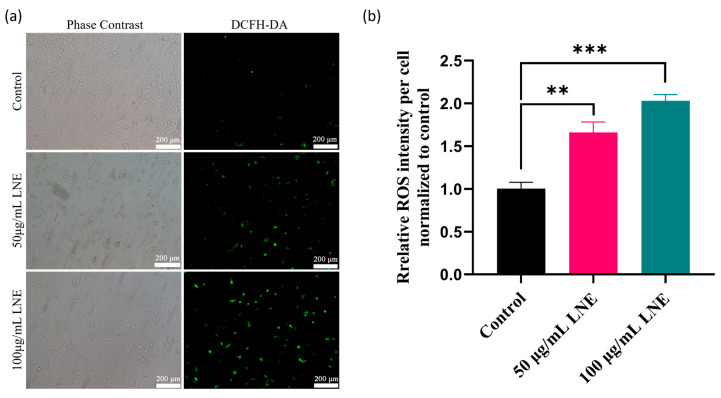
LNE induces intracellular reactive oxygen species (ROS) accumulation in MDA-MB-231 breast cancer cells. (**a**) Fluorescence microscopy images of DCFH-DA-stained MDA-MB-231 cells after treatment with LNE (50 and 100 μg/mL for 48 h), showing increased green fluorescence intensity indicative of ROS production; (**b**) quantitative analysis of mean fluorescence intensity of DCF signal. LNE treatment significantly elevated ROS levels in a dose-dependent manner compared to the control group. Data are presented as the mean ± SD (*n* = 3). ** *p* < 0.01, *** *p* < 0.001 vs. control.

Cell signaling is mainly regulated by the amount of ROS produced within the cytoplasm. We conducted an analysis using the fluorescent probe 2′,7′-dichlorodihydrofluorescein diacetate (DCFH-DA) with fluorescence microscopy. DCFH-DA is a fluorescent probe that has no fluorescence itself but is lipophilic and can freely pass through the cell membrane. After entering the cell, it can be hydrolyzed by intracellular esterases to generate DCFH. DCFH cannot permeate the cell membrane, making it easy for the probe to be loaded into the cell. Intracellular ROS can oxidize the non-fluorescent DCFH to generate the strongly fluorescent substance 2′,7′-dichlorofluorescein (DCF), and its fluorescence intensity is directly proportional to the level of intracellular ROS. By detecting the fluorescence of DCF, the level of intracellular ROS can be known. Compared with the low level of green light emitted by the control MDA-MB-231 cells, the DCFH-DA analysis demonstrated that LNE induced a higher level of ROS in the treated MDA-MB-231 cells (Figure 5).

**Figure 6:**
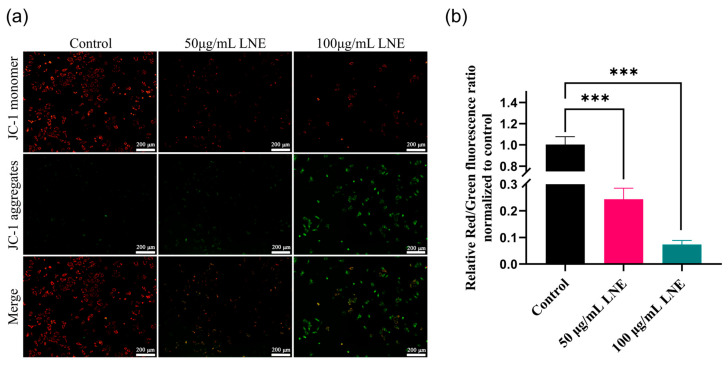
Effect of LNE on mitochondrial membrane potential (ΔΨm) in MDA-MB-231 cells. (**a**) JC-1 staining images of MDA-MB-231 cells treated with LNE (50 and 100 μg/mL, 48 h), showing a concentration-dependent shift from red fluorescence (JC-1 aggregates, normal ΔΨm) to green fluorescence (JC-1 monomers, depolarized ΔΨm), indicating mitochondrial depolarization; (**b**) quantitative analysis of the red/green fluorescence intensity ratio. LNE treatment significantly reduced mitochondrial membrane potential, suggesting early-stage apoptosis induction. Data are presented as the mean ± SD (*n* = 3). *** *p* < 0.01 vs. control.

Since mitochondria are the main organelles that control cellular bioenergy, they also play a crucial role in the homeostasis of ROS and the intrinsic pathway of apoptosis. The decrease in mitochondrial membrane potential is a hallmark event in the early stage of apoptosis. JC-1 is an ideal fluorescent probe widely used for detecting the mitochondrial membrane potential (∆Ψm). When the mitochondrial membrane potential is high, JC-1 aggregates in the mitochondrial matrix to form polymers (JC-1 aggregates), which can produce red fluorescence. When the mitochondrial membrane potential is low, JC-1 cannot aggregate in the mitochondrial matrix. JC-1 exists as a monomer (JC-1 monomer) and can produce green fluorescence. Through the JC-1 detection assay, the decrease in the cell membrane potential can be easily detected by the transition from red fluorescence to green fluorescence, which serves as an indicator for the early stage of apoptosis. Figure 6 shows that in the MDA-MB-231 cells of the control group, the mitochondrial membrane potential (MMP) is high and there is bright red fluorescence. However, after treatment with LNE, the apoptosis of MDA-MB-231 cells is induced. In apoptotic cells with low MMP, JC-1 remains in the monomeric form and exhibits green fluorescence.

### 3.8. LNE-Induced Apoptosis Is Mediated by ROS Production

**Figure 7:**
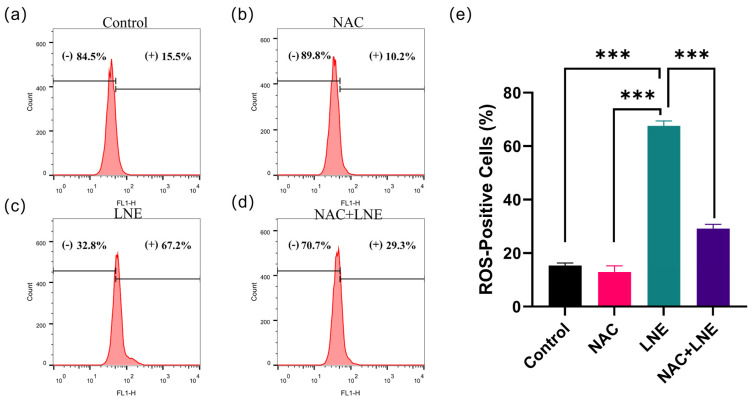
NAC pre-treatment effectively abrogates LNE-induced ROS accumulation. MDA-MB-231 cells were pre-treated with or without 5 mM N-acetylcysteine (NAC) for 2 h, followed by treatment with LNE (50 μg/mL) for 48 h. Intracellular ROS levels were measured using the DCFH-DA probe followed by flow cytometry. (**a**–**d**) Representative histograms showing the fluorescence intensity of DCF in (**a**) control, (**b**) NAC alone, (**c**) LNE alone, and (**d**) NAC+LNE groups. The gate (+) indicates the percentage of ROS-positive cells. (**e**) Quantification of the percentage of ROS-positive cells. Data are presented as the mean ± SD from three independent experiments. *** *p* < 0.001.

To determine whether the elevation of intracellular ROS is a direct cause of LNE-induced apoptosis, we conducted a rescue experiment using the ROS scavenger N-acetylcysteine (NAC). We first confirmed that LNE treatment significantly increased the percentage of ROS-positive MDA-MB-231 cells from 15.3% (control) to 67.5%. As anticipated, pre-treatment with NAC effectively abrogated this LNE-induced ROS accumulation, with the level of ROS-positive cells returning to near-control levels (Figure 7).

**Figure 8:**
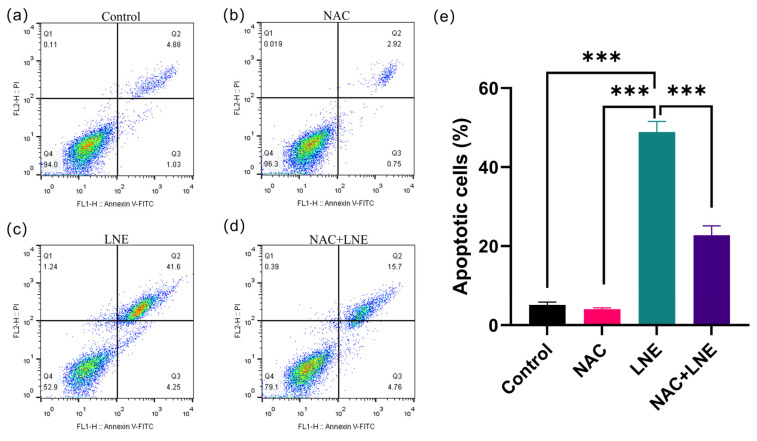
LNE-induced apoptosis in MDA-MB-231 cells is dependent on ROS generation. MDA-MB-231 cells were treated under the same conditions as described in Figure 7. Cell apoptosis was assessed by Annexin V-FITC/PI staining and flow cytometry. (**a**–**d**) Representative flow cytometry plots for the (**a**) control, (**b**) NAC alone, (**c**) LNE alone, and (**d**) NAC+LNE groups. The quadrants represent live cells (Q4, Annexin V−/PI−), early apoptotic cells (Q3, Annexin V+/PI−), and late apoptotic/necrotic cells (Q2, Annexin V+/PI+). (**e**) Quantification of the total apoptosis rate (Q2 + Q3). Data are presented as the mean ± SD from three independent experiments. *** *p* < 0.001.

Next, we investigated whether scavenging ROS could protect cells from apoptosis. Consistent with our earlier findings, LNE treatment markedly increased the total apoptosis rate to 48.8% (Figure 8). Strikingly, the pro-apoptotic effect of LNE was significantly attenuated in the presence of NAC, with the apoptosis rate decreasing to 22.7%. Treatment with NAC alone had no significant effect on either basal ROS levels or cell apoptosis.

Collectively, these findings provide direct evidence that the accumulation of intracellular ROS is a critical upstream mechanism driving LNE-induced apoptosis in MDA-MB-231 cells.

### 3.9. Effect of LNE on the Migration of MDA-MB-231 Cells Determined by Scratch Assay

**Figure 9:**
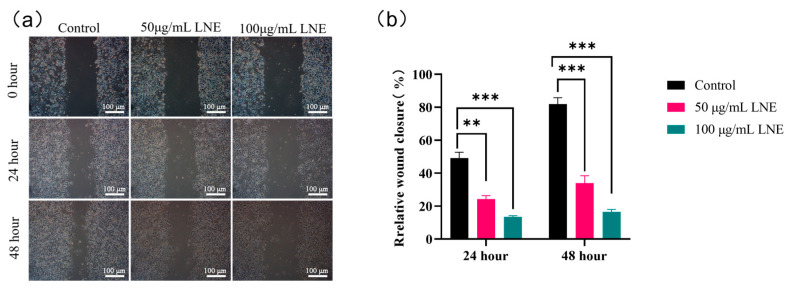
Anti-migration effect of LNE in MDA-MB-231 cells. (**a**) Representative images of wound healing (scratch) assay showing reduced migration of MDA-MB-231 cells following treatment with LNE (50 and 100 μg/mL) for 0, 24, and 48 h; (**b**) quantitative analysis of wound closure area at 24- and 48-h post-treatment. LNE treatment significantly inhibited cell migration in a time- and dose-dependent manner compared with the control (0.1% DMSO). Data are presented as the mean ± SD of three independent experiments. ** *p* < 0.01, *** *p* < 0.001 vs. control.

To specifically assess the effect of LNE on cell migration while minimizing confounding effects from cell proliferation, a wound healing assay was performed under serum-free conditions. The effect of LNE on the migration of MDA-MB-231 cells was evaluated using the wound healing assay. When monitored every 24 h, the untreated cells underwent proliferation and migration. LNE reduced the migration of MDA-MB-231 cells in a dose- and time-dependent manner. After 48 h of treatment, the relative wound closure percentage of the untreated control cells reached 81.92%. As shown in Figure 9, the relative wound closure percentages at the treatment concentrations of 50 and 100 μg/mL were 33.94% and 16.55%, respectively.

### 3.10. In Vivo Studies

#### 3.10.1. Effect of LNE on the Original 4T1 Breast Tumor Burden in Mice

**Figure 10:**
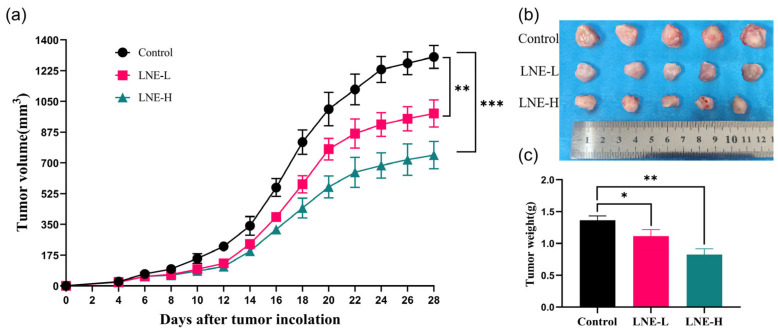
LNE suppresses tumor growth in a 4T1 orthotopic breast cancer mouse model. (**a**) Tumor growth curves of BALB/c mice bearing 4T1 tumors treated with LNE at 100 and 300 mg/kg/day over the experimental period; (**b**) representative images of excised tumors from each group at the experimental endpoint; (**c**) final tumor weights measured after sacrifice. LNE treatment significantly reduced tumor volume and weight compared with the control group. Data are presented as the mean ± SD (*n* = 5). * *p* < 0.05, ** *p* < 0.01, *** *p* < 0.001 vs. control.

The promising findings from the in vitro studies provided a basis for further exploring the therapeutic potential of LNE in the in vivo system. All BALB/c mice inoculated with 4T1 breast cancer cells had palpable tumors on the 4th day after inoculation. Treatment with LNE was initiated on the 4th day, and the volume of the subcutaneous tumors was measured every two days using a vernier caliper. Over time, the tumor volume in the mice gradually increased. Compared with the control group, the treatment with LNE could regress the tumor burden (Figure 10a). After 24 days of LNE treatment, the mice were sacrificed, and the tumors from each group were excised and weighed. Compared with the control group, the treatment with LNE could significantly regress the tumor burden. There were significant differences in the tumor weights between the high and low-dose LNE groups and the control group. The tumor inhibition rates of the LNE-L group and the LNE-H group were 42.90% (*p* < 0.05) and 24.71% (*p* < 0.01), respectively (Figure 10c).

#### 3.10.2. Effect of LNE on the Morphology of Tumor Tissues and Metastatic Tissues

**Figure 11:**
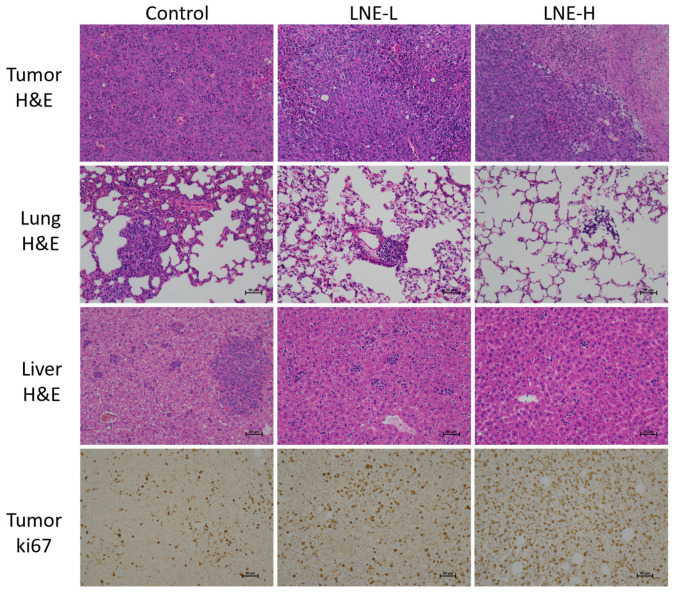
Representative images of hematoxylin and eosin (H&E) and Ki67-stained tissues from control and LNE-treated mice. The top row shows H&E staining of primary tumor tissues, revealing increased necrosis in the LNE-treated groups. The second and third rows show H&E staining of lung and liver tissues, respectively, indicating reduced metastatic nodules in LNE-treated groups. The bottom row shows immunohistochemical staining for Ki67 in tumor tissues, indicating decreased cell proliferation upon LNE treatment. Original magnification, 200×. Scale bars = 100 µm.

By examining the H&E staining of tumor tissues, and lung and liver tissues, it was observed that in the tumor tissues of the control group, the tumor cells were closely arranged, with obvious nucleoli, deep staining, and basically no necrosis. In contrast, in the LNE-L group and the LNE-H group, the tumor cells were loosely arranged, with blurred cell contours, ruptured cytoplasm, and a larger necrotic area of tumor cells, suggesting that LNE has an anti-breast cancer effect (Figure 11).

The liver and lung tissues of the control group showed tumor metastasis and infiltration of immune cells, while the number of metastatic nodules was reduced in the LNE treatment groups. In the control group, there were a large number of metastatic cancer cells in the lungs, with an adenoid arrangement, and the alveolar structure was almost invisible. Both treatment groups alleviated the pathological changes of the lung structure and the lung metastasis of cancer cells. The liver of the mice in the control group was filled with a large number of scattered metastatic lesions, with large nuclei and deep staining. In comparison, the proportion of metastatic cancer cells in the two treatment groups was significantly reduced. These findings indicate that LNE inhibits the growth and metastasis of TNBC. In addition, ki67 staining showed that the apoptosis rate in the LNE-H group increased significantly. These results confirm that LNE exerts an anti-tumor effect by promoting apoptosis and simultaneously reduces the metastasis of TNBC.

#### 3.10.3. Identification of the Blood-Absorbed Constituents of LNE via HPLC-Q-TOF-MS

Analyzing the components in the serum can provide a deeper understanding of the pharmacological mechanism of herbal medicine. The serum pharmacology theory of herbal medicine holds that the components absorbed into the blood are the active ingredients through which the herbal medicine exerts its effects. In this work, HPLC-Q-TOF-MS was used for the qualitative analysis of the prototype chemical components that migrated into the serum after mice ingested LNE. The total ion chromatograms (TICs) in the positive ionization and negative ionization modes are shown in Figure S2. After comparison with pure LNE and blank serum, 65 compounds of LNE that could be absorbed into the serum were identified in the serum samples of mice (Table S1).

#### 3.10.4. Screening of Anti-Breast Cancer Targets of *Lobelia nummularia* Ethanol Extract

**Figure 12:**
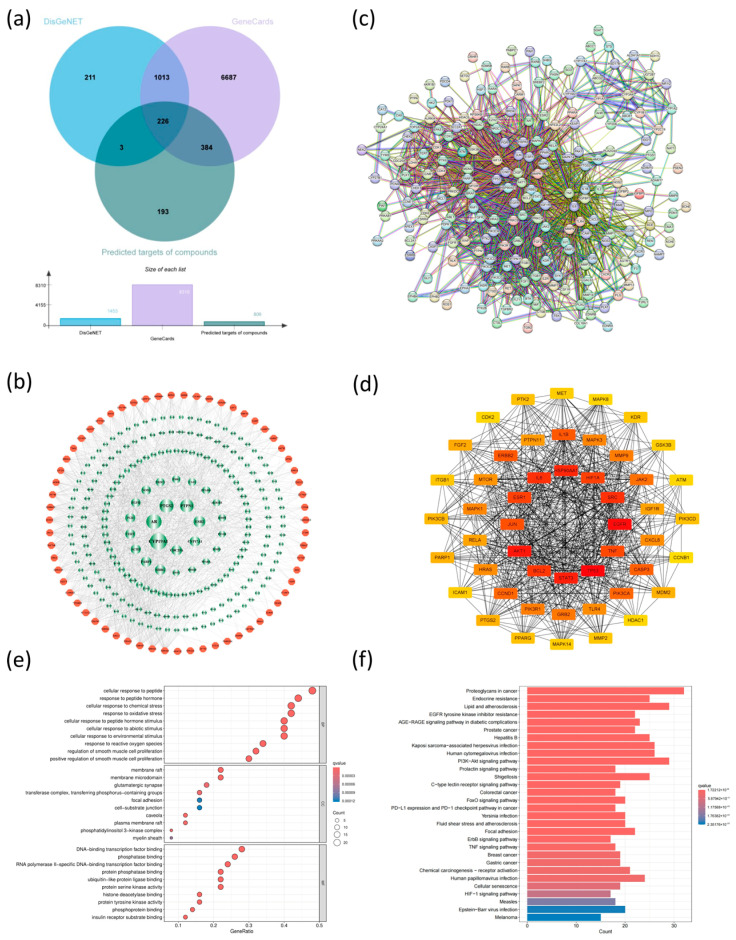
The pharmacology-based prediction of anti-breast cancer mechanisms of LNE. (**a**) Venn diagram showing overlapping targets between LNE-absorbed compounds and breast cancer-related genes. (**b**) Compound–target network illustrating interactions between absorbed compounds (orange octagons) and breast cancer targets (red circles; larger size indicates higher degree values). (**c**) Protein–protein interaction (PPI) network of key therapeutic targets. (**d**) Top 50 hub genes identified by degree analysis. (**e**) GO enrichment analysis highlighting biological processes, cellular components, and molecular functions involved. (**f**) KEGG pathway enrichment analysis indicating critical pathways such as PI3K-Akt, EGFR resistance, TNF signaling, and HIF-1 signaling.

To further explore the mechanism of LNE treatment, network pharmacology was used to predict the potential molecular mechanism. By searching for the 65 components in the LNE-containing serum in the Swiss Target Prediction database, a total of 806 potential targets were obtained. In addition, 8310 targets retrieved from Gene Cards and 1453 targets retrieved from the Disgenet database were selected as the targets of breast cancer, respectively. By constructing a Venn diagram to intersect the component targets with the disease targets, 226 overlapping targets were obtained and identified as the therapeutic targets of LNE in the treatment of anti-breast cancer (Figure 12a, Table S2).

#### 3.10.5. Network Construction and Topological Analysis

A component–disease–target network for the treatment of breast cancer by LNE was established (Figure 12b). The PPI network of 226 targets of LNE in the treatment of breast cancer was developed through the STRING database (Figure 12c). These results indicate that the anti-breast cancer effect of LNE has the characteristic of multiple targets. At the same time, significant correlations were observed among the target proteins. To further focus on the core set of target genes, we focused on the genes with the top 50 degree values in the subsequent analysis (Figure 12d).

#### 3.10.6. GO and KEGG Enrichment Analysis

To elucidate the biological functions and signaling pathways associated with the top 50 candidate targets, Gene Ontology (GO) and Kyoto Encyclopedia of Genes and Genomes (KEGG) enrichment analyses were performed. A total of 2320 biological process (BP) terms, 56 cellular component (CC) terms, and 149 molecular function (MF) terms met the significance threshold of *p* < 0.05. The top 10 significantly enriched terms in each GO category are shown in Figure 12e. In the BP category, enriched terms were primarily related to response to oxidative stress and other key processes. CC enrichment included terms such as membrane raft and phosphatidylinositol 3-kinase complex, while MF terms were mainly associated with DNA-binding transcription factor binding and related functions. Additionally, KEGG analysis revealed 169 significantly enriched pathways (*p* < 0.05). As shown in Figure 12f, the anticancer mechanisms of LNE in breast cancer are primarily associated with the PI3K-Akt signaling pathway, EGFR tyrosine kinase inhibitor resistance, TNF signaling pathway, and HIF-1 signaling pathway.

### 3.11. Molecular Docking Analysis of Key Compound-Target Interactions

**Figure 13:**
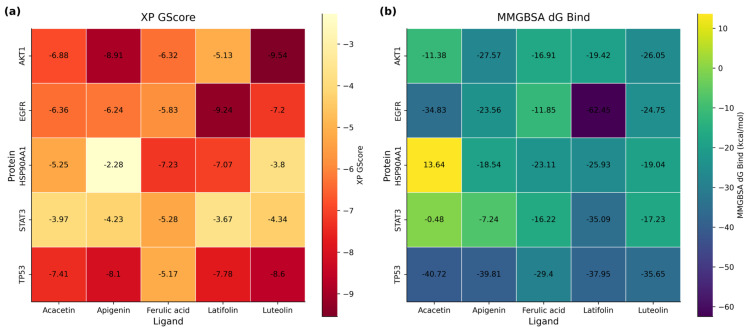
Molecular docking and binding free energy analysis of representative compounds in LNE with key breast cancer-related targets. (**a**) XP GlideScore (XP GScore) heatmap showing the docking scores of five representative ligands (acacetin, apigenin, ferulic acid, latifolin, luteolin) against five core protein targets (AKT1, EGFR, HSP90AA1, STAT3, TP53). A more negative score indicates stronger predicted binding affinity. (**b**) MMGBSA ΔG Bind heatmap representing calculated binding free energies (kcal/mol) of the same ligand–protein pairs. Lower values (more negative) suggest greater binding stability. Notably, latifolin and luteolin exhibited strong predicted interactions with EGFR and TP53, suggesting their potential role in modulating key pathways in breast cancer.

Molecular docking was used to identify the molecular interactions between the active compounds in LNE and the target proteins. Generally, this model evaluates the free energy of the interaction between the ligand and the receptor. According to the degree values in the component–disease–target network, the main active components in the LNE—containing serum were likely to be ferulic acid, apigenin, acacetin, luteolin, and latifolin. Meanwhile, TP53, EGFR, AKT1, STAT3, and HSP90AA1 were the key targets predicted in the PPI network. Based on these network pharmacology results, molecular docking was carried out to explore the interactions between these compounds as ligands and the target proteins. In this study, the binding characteristics of five proteins and five ligands were evaluated by two indexes, XP GScore and MM—GBSA dG Bind. It is generally believed that if the XP GScore is less than −6, it indicates that the ligand has stable binding properties with the protein, and if the MM-GBSA dG Bind value is lower than −25 kcal/mol, it indicates that the binding free energy is low and the ligand binds stably to the protein. As shown in the heat map of the docking results (Figure 13), XP GScore showed that AKT1 had the strongest binding with luteolin (−9.544). In addition, apigenin-AKT1, acacetin-EGFR, latifolin-EGFR, ferulic acid-HSP90AA1, latifolin-HSP90AA1, luteolin-TP53, apigenin-TP53, acacetin-TP53, and latifolin-TP53 all had strong binding interactions between the components and the target proteins.

**Figure 14:**
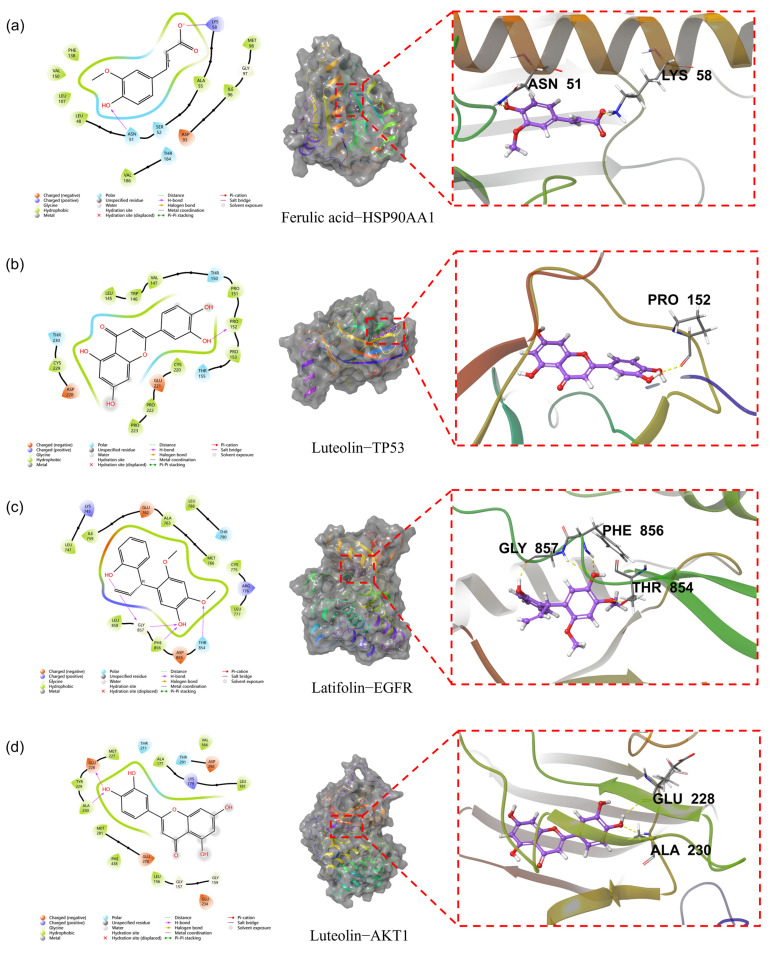
Molecular docking models of key compounds binding to breast cancer targets. (**a**) Ferulic acid bound to HSP90AA1 (PDB ID: 5J2X); (**b**) luteolin bound to TP53 (PDB ID: 6GGC Chain A); (**c**) latifolin bound to EGFR (PDB ID: 8A27); (**d**) luteolin bound to AKT1 (PDB ID: 4GV1). Hydrogen bonds, salt bridges, and hydrophobic interactions stabilized the ligand–protein complexes, providing structural evidence for the bioactivity of LNE components.

The lower the binding energy, the stronger the predicted interaction, suggesting potential key compound–target associations for anti-breast cancer effects. Hydrogen bonds, salt bridges, and hydrophobic interactions formed by the binding of small molecular compounds to proteins are crucial for the stability of the complex structure. Images of the ligand–protein docking complex, molecular surface, and 2D/3D interactive diagrams are shown in Figure 14.

### 3.12. LNE Suppresses the EGFR/PI3K/AKT/mTOR Pathway and Activates the TP53-Mediated Apoptotic Cascade In Vivo

**Figure 15:**
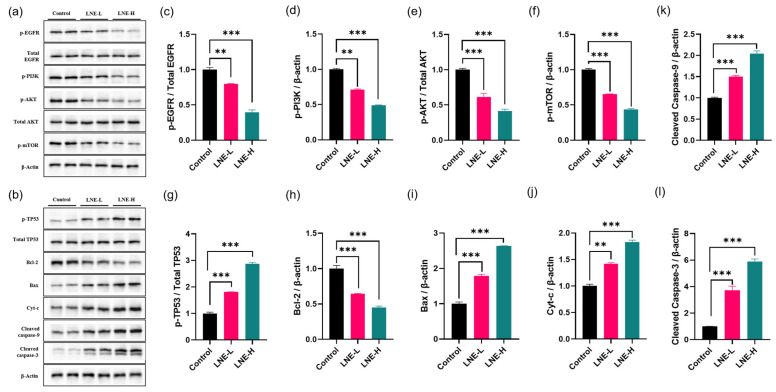
LNE suppresses the EGFR/PI3K/AKT/mTOR pathway and activates the TP53-mediated apoptotic cascade in vivo. The effect of LNE on key signaling and apoptosis-related proteins was assessed in 4T1 tumor tissues by Western blot. (**a**) Representative Western blot images showing the expression levels of key proteins in the PI3K/AKT/mTOR signaling pathway. (**b**) Representative blot images showing the expression levels of key proteins in the TP53-mediated apoptosis pathway. (**c**–**l**) Densitometric quantification of the protein bands shown in (**a**,**b**). The expression of phosphorylated proteins (p-EGFR, p-AKT, p-TP53) was normalized to their respective total proteins. The expression of other proteins was normalized to β-actin. Data are presented as the mean ± SD (*n* = 3). ** *p* < 0.01, *** *p* < 0.001 compared to the control group.

To elucidate the molecular mechanisms underlying the in vivo antitumor efficacy of LNE, we examined the expression and phosphorylation status of key proteins in survival and apoptosis pathways in 4T1 tumor tissues by Western blot (Figure 15).

We first investigated LNE’s effect on the PI3K/AKT/mTOR signaling cascade. As shown in Figure 15a,c–f, LNE treatment caused a dose-dependent decrease in the phosphorylation of EGFR, PI3K, AKT, and mTOR. Importantly, the expression levels of total EGFR and total AKT remained constant across all groups. The quantification of the p-EGFR/total EGFR and p-AKT/total AKT ratios confirmed that LNE significantly inhibits the activation of this pathway, rather than altering total protein expression. This demonstrates that LNE effectively suppresses this critical pro-survival signaling cascade in vivo.

Concurrently, we assessed proteins involved in the TP53-mediated mitochondrial apoptosis pathway (Figure 15b,g–l). LNE treatment significantly increased the phosphorylation of TP53, while the level of total TP53 was unaffected, indicating its activation. This was accompanied by a pro-apoptotic shift in the Bax/Bcl-2 ratio. Furthermore, we observed elevated levels of cytochrome c and, crucially, a significant upregulation of both the initiator cleaved caspase-9 and the final executor cleaved caspase-3.

Taken together, these molecular data demonstrate that LNE exerts its antitumor effect in vivo by suppressing the PI3K/AKT/mTOR survival pathway, while concomitantly activating the full mitochondrial apoptotic cascade, from TP53 activation down to the final execution step of caspase-3 cleavage.

## 4. Discussion

In the present study, we conducted a comprehensive investigation into the anticancer mechanisms of the traditional medicinal herb *Lobelia nummularia* against breast cancer. Our findings provide, for the first time, a robust body of evidence demonstrating that its ethanolic extract (LNE) exerts potent antitumor effects in vitro and in vivo. The central finding of our work is that LNE induces ROS-dependent mitochondrial apoptosis in breast cancer cells. This pro-apoptotic activity is driven by a dual mechanism: the suppression of the pro-survival EGFR/PI3K/AKT signaling pathway and the activation of the TP53-mediated apoptotic cascade. Furthermore, we established a clear phytochemical basis for these effects by quantifying key bioactive flavonoids within the extract.

A fundamental challenge in the pharmacological study of herbal extracts is attributing the observed biological effects to specific chemical constituents within a complex mixture. Our study addressed this by moving beyond qualitative profiling to the quantitative analysis of five key flavonoids, including luteolin and apigenin, which were prioritized by our in silico analysis. The confirmation of their substantial presence in LNE (Table 2) provides a clear phytochemical foundation for its bioactivity.

This finding is highly significant, as these quantified compounds are well-documented anticancer agents. For instance, luteolin has been extensively reported to induce apoptosis and cell cycle arrest in various breast cancer models, often through modulation of the PI3K/AKT pathway [22,23]. Similarly, apigenin is a known inhibitor of cancer cell proliferation and migration, and has been shown to sensitize cancer cells to conventional therapies [24,25,26]. The presence of other quantified flavonoids like acacetin and ferulic acid further contributes to the extract’s potential synergistic effects. Therefore, our quantitative data, in conjunction with the established anticancer profiles of these individual flavonoids, strongly support the hypothesis that they are major contributors to the overall therapeutic efficacy of the LNE extract.

Beyond identifying its chemical constituents, a central goal of this study was to elucidate the primary mechanism driving LNE’s anticancer effects. Our findings compellingly identify the induction of oxidative stress as this critical initiating event. While our initial experiments revealed a strong correlation between LNE treatment, elevated intracellular ROS, and apoptotic cell death, the causal relationship was unequivocally established by our NAC rescue experiment.

The demonstration that pre-treatment with the antioxidant NAC could significantly reverse LNE-induced apoptosis (Figure 8) provides direct evidence that ROS generation is an essential mediator, rather than a mere consequence, of LNE’s pro-apoptotic activity. This mechanism positions LNE within a well-established class of anticancer agents that function by modulating cellular redox balance. Cancer cells often exhibit higher basal ROS levels and a compromised antioxidant capacity, rendering them more vulnerable to further oxidative insults [27]. By elevating ROS beyond a tolerable threshold, LNE appears to selectively push cancer cells toward apoptosis, a strategy employed by various successful natural chemotherapeutics, such as paclitaxel [28,29].

Thus, the ROS-dependent apoptotic pathway serves as the foundational mechanism that likely precedes and orchestrates the downstream modulation of the signaling pathways observed in our study.

Our study further delineated the downstream molecular signaling cascades orchestrated by LNE-induced oxidative stress. The PI3K/AKT/mTOR pathway is a central regulator of cell survival and is frequently hyperactivated in breast cancer, making it a prime therapeutic target [9,30]. Our Western blot analysis, normalized against total protein levels, conclusively demonstrates that LNE treatment leads to a significant downregulation of EGFR, PI3K, AKT, and mTOR phosphorylation (Figure 15). This potent suppression of a critical pro-survival axis is a key component of LNE’s antitumor effect.

Concomitantly, LNE triggered the activation of the TP53 tumor suppressor pathway. The increased phosphorylation of TP53, along with an elevated Bax/Bcl-2 ratio, initiated the mitochondrial apoptotic program. Importantly, our confirmation of the subsequent cleavage of both initiator caspase-9 and the final executor caspase-3 provides unequivocal evidence for the engagement of the full intrinsic apoptotic cascade. It is well-established that oxidative stress can act as a potent trigger for TP53 activation and can also disrupt receptor tyrosine kinase signaling like EGFR [31,32]. Thus, we propose a cohesive mechanistic model where LNE-induced ROS accumulation acts as a pivotal stress signal that delivers a “double-hit” to cancer cells: it simultaneously deactivates the PI3K/AKT survival engine while activating the TP53-driven apoptotic machinery, leading to efficient cell death.

In addition to our functional analyses, we employed molecular docking to explore a plausible structural basis for LNE’s multi-target effects. We acknowledge that in silico docking is inherently a hypothesis-generating tool. To enhance confidence in our predictions, we first validated our docking protocol via a representative re-docking experiment, which confirmed its reliability (RMSD < 2.0 Å). Our docking results revealed high-affinity interactions between key flavonoids and the signaling proteins investigated in this study (Figure 13). For instance, the predicted binding of compounds like latifolin to the active site of EGFR provides a rational structural explanation for our experimental finding that LNE potently inhibits EGFR phosphorylation. Similarly, the strong predicted interaction between other components like luteolin and AKT1 aligns with the observed suppression of the PI3K/AKT pathway. Furthermore, our quantitative HPLC analysis adds another layer of validation by confirming that these specific flavonoids are substantially present within the LNE extract, making these molecular interactions biologically plausible.

While these in silico findings are not a substitute for direct binding or enzymatic assays, they integrate seamlessly with our experimental data. Together, the quantitative phytochemical data, the validated docking predictions, and the functional pathway analysis provide a cohesive, multi-layered model supporting a multi-component, multi-target mechanism of action for LNE.

Furthermore, our molecular docking studies were performed using human protein structures, while the in vivo validation was in a murine model. To address this potential discrepancy, we performed a sequence alignment of the human and murine orthologs for our key targets (EGFR, AKT1, TP53). The analysis revealed a high degree of homology (>95% identity), particularly within critical ligand-binding domains. This strong conservation across species provides confidence that the binding modes predicted for the human proteins are highly relevant to the murine system.

Despite the robust findings presented, it is important to acknowledge the limitations of this study, which also highlight promising avenues for future research. First, our investigation focused on the whole ethanolic extract of LNE to validate its traditional use. While we successfully quantified several key components, future work should involve bioactivity-guided fractionation to isolate the most potent individual compounds and elucidate their specific mechanisms and potential synergies. Second, while our data strongly support the functional modulation of the EGFR/PI3K/AKT and TP53 pathways, we did not perform direct enzymatic or biophysical binding assays. Such experiments will be crucial for definitively confirming the compound-target interactions predicted by our docking analysis. Finally, our in vivo study established the standalone efficacy of LNE, but did not include a positive control group for comparative purposes. Future studies should aim to benchmark LNE’s potency against standard-of-care chemotherapeutics and explore its potential in combination therapies to enhance efficacy and reduce toxicity.

Additionally, our study used the human MDA-MB-231 cell line for in vitro mechanistic work and the murine 4T1 line for the in vivo syngeneic model. This approach was chosen strategically, as MDA-MB-231 is a gold-standard model for human TNBC mechanistic studies, while the 4T1 syngeneic model is indispensable for evaluating efficacy and metastasis in an immunocompetent host. Importantly, both cell lines share key features; they are both considered models of the highly aggressive claudin-low subtype of TNBC, suggesting a degree of molecular and phenotypic similarity. Our cytotoxicity data further supports this, confirming that both cell lines exhibit a comparable high sensitivity to LNE treatment in vitro (Figure 3). Nevertheless, we acknowledge that TNBC is a heterogeneous disease, and future studies involving other subtypes would be beneficial to broaden the applicability of our findings.

## 5. Conclusions

In conclusion, this study provides a comprehensive validation of the anticancer effects of LNE against breast cancer. We demonstrated, for the first time, that LNE’s primary mechanism of action is the induction of ROS-dependent mitochondrial apoptosis. This is achieved through a dual strategy of suppressing the pro-survival EGFR/PI3K/AKT pathway while simultaneously activating the TP53-mediated apoptotic cascade. Furthermore, we established a clear phytochemical basis for this activity by quantifying key bioactive flavonoids, such as luteolin and apigenin, within the extract. Collectively, our findings position LNE not just as a promising traditional remedy, but also as a mechanistically well-defined natural product candidate for the development of novel breast cancer therapeutics.

## Figures and Tables

**Table 1:** Chemical characteristics of ethanolic extracts from *Lobelia nummularia* by UPLC-Q-TOF.

No.	Name	Formula	PubChem CID	Experimental Mass	Retention Time (s)	Error (ppm)	ms2 Adduct	Fragment Ions
1	Stachydrine	C_7_H_13_NO_2_	115244	144.1020015	31.5758	0.010537654	[M+H]+	144.101146; 84.080841; 58.065159; 126.128348; 119.419592
2	Luteolin 6-C-glucoside 8-C-arabinoside	C_27_H_30_O_16_	3549960	609.1474678	44.0334	2.409582862	[M−H]−	609.148219; 369.060713; 399.068383; 489.108606; 339.051556
3	Mecamylamine	C_11_H_21_N	4032	168.1747615	50.00785	1.418221913	M+H	168.174269; 96.08015; 169.036027; 98.09618; 81.070174
4	KOBUSONE	C_14_H_22_O_2_	6710676	240.1957444	55.354	1.063924211	[M+NH_4_]+	240.195169; 116.107206; 58.065149; 168.138912; 96.080923
5	Luteolin 7-diglucuronide	C_27_H_26_O_18_	5282153	637.1034196	56.5514	2.480547191	M−H	285.041504; 637.107566; 351.056026; 113.024071; 85.029135
6	Isoviolanthin	C_27_H_30_O_14_	101422758	577.15554	92.2036	0.797083227	M−H	577.159142; 353.066993; 383.07698; 457.111106; 96.969484
7	Complanatuside	C_28_H_32_O_16_	5492406	669.1661464	98.08	2.770017312	M+HCOO	299.055642; 461.104242; 284.030824; 285.041273; 92.680996
8	Kaempferol-7-O-neohesperidoside	C_27_H_30_O_15_	22178437	593.1517476	104.523	0.425453913	[M−H]−	285.041179; 593.145822; 284.031119; 65.908963; 113.025004
9	coniferin	C_16_H_22_O_8_	5280372	341.1240745	110.0625	0.218302657	[M−H]−	59.013955; 89.02417; 161.061407; 71.013681; 119.035053
10	Ferulic acid	C_10_H_10_O_4_	445858	195.0652329	110.538	1.193821788	[M+H]+	177.053943; 145.029184; 117.033485; 149.060482; 137.059385
11	Fraxidin	C_11_H_10_O_5_	3083616	223.0600451	119.282	0.202181336	M+H	223.061278; 190.02662; 208.035693; 107.048879; 163.038906
12	Riboprine	C_15_H_21_N_5_O_4_	24405	336.1666443	124.828	1.058079384	M+H	204.124472; 136.06205; 336.170314; 148.061031; 161.095865
13	5,7-Dihydroxychromone	C_9_H_6_O_4_	5281343	179.0340958	130.096	0.535338091	M+H	179.034489; 133.10047; 105.070142; 119.085012; 161.096
14	Scolymoside	C_27_H_30_O_14_	5282152	579.1694132	136.458	2.739837438	[M+H]+	271.05789; 433.108463; 85.02838; 71.048962; 579.161749
15	Pinoresinol 4-O-β-D-glucopyranoside	C_26_H_32_O_11_	486614	519.1879002	144.175	1.733783223	M−H	151.040621; 357.134574; 136.01692; 342.111936; 71.013654
16	Pinoresinol	C_20_H_22_O_6_	73399	341.1386165	144.197	1.807099734	M+H-H_2_O	137.059485; 291.100616; 341.194003; 323.127251; 271.094876
17	Genistein	C_15_H_10_O_5_	5280961	271.0593836	145.892	2.273908332	[M+H]+	271.06211; 153.017829; 119.049142; 92.655186; 272.063805
18	Cynaroside	C_21_H_20_O_11_	5280637	447.092682	151.141	0.711158319	M−H	285.041211; 447.093001; 286.043464; 284.031065; 92.680147
19	Rupestonic acid	C_15_H_20_O_3_	24094149	249.1475434	161.996	6.194885616	M+H	249.147868; 92.657727; 203.143353; 81.069482; 145.100533
20	Beta-Ionone	C_13_H_20_O	638014	193.1588105	170.629	0.981050279	M+H	193.157839; 135.11705; 109.100915; 175.147548; 133.100598
21	DiosMetin 7-O-β-D-Glucuronide	C_22_H_20_O_12_	163006812	477.1027193	170.724	0.588367273	M+H	301.072533; 286.047122; 477.105174; 258.05208; 53.010567
21	Diosmetin	C_16_H_12_O_6_	5281612	301.070716	269.096	0.943328686	[M+H]+	301.072595; 286.047098; 258.052202; 81.069478; 95.08489
22	Lobetyolin	C_20_H_28_O_8_	53486204	441.1766452	181.792	1.462452492	[M+FA]-	185.097802; 89.024214; 143.070626; 59.014012; 159.081211
23	Spinosin	C_28_H_32_O_15_	155692	607.1672125	194.127	0.349939428	M−H	283.061329; 607.171074; 268.038726; 67.466445; 78.95929
24	Tuberostemonine	C_22_H_33_NO_4_	100781	376.2479048	201.319	0.25294059	[M+H]+	376.248801; 377.247515; 358.233612; 147.103664; 261.174318
25	Fraxinellone	C_14_H_16_O_3_	124039	215.1067854	203.194	0.997819514	M+H-H_2_O	215.105705; 145.065711; 155.085119; 187.111516; 169.100661
26	Usaramine	C_18_H_25_NO_6_	5281756	352.1726306	205.963	1.048932421	M+H	352.170472; 295.150637; 92.657724; 98.096297; 168.174312
27	Luteolin	C_15_H_10_O_6_	5280445	287.0551804	209.087	0.628452748	M+H	287.05467; 153.018018; 135.044122; 288.058286; 117.032437
28	Atractylenolide II	C_15_H_20_O_2_	14448070	233.1536143	209.134	1.654448966	[M+H]+	233.154667; 71.048887; 105.070182; 131.084822; 91.054277
29	Latifolin	C_17_H_18_O_4_	340211	287.1249013	213.75	0.343592741	[M+H]+	287.054684; 153.018255; 135.044133; 288.058309; 270.150185
30	Linarin	C_28_H_32_O_14_	5317025	615.1685984	223.922	0.972718738	M+Na	615.169949; 307.055942; 331.100059; 68.351735; 493.124227
31	(2S,3R,4S)-4-Hydroxyisoleucine	C_6_H_13_NO_3_	6918732	130.0863195	226.623	2.456282791	M+H-H_2_O	84.044273; 130.064604; 107.950323; 71.929408; 125.960848
32	Methyl 4-hydroxycinnamate	C_10_H_10_O_3_	5319562	177.0555744	230.9275	2.403598183	[M−H]−	177.054427; 145.029807; 117.034873; 121.029215; 118.043024
33	Acacetin	C_16_H_12_O_5_	5280442	285.0747007	243.296	1.049777021	M+H	285.076868; 270.053533; 242.056158; 92.656033; 286.079336
34	Asiaticoside	C_48_H_78_O_19_	108062	1003.510537	246.35	0.535444462	M+HCOO	487.347446; 957.508084; 957.479987; 469.151768; 101.024141
35	Ergolide	C_17_H_22_O_5_	185786	324.1781173	248.736	0.361924876	M+NH_4_	324.177938; 211.094179; 98.096178; 92.656032; 154.082545
36	Syringaresinol	C_22_H_26_O_8_	332426	419.1676828	252.173	0.756695406	[M+H]+	419.168493; 132.065422; 72.044052; 257.114948; 254.958445
37	Kavain	C_14_H_14_O_3_	5281565	213.0910271	259.4415	0.126984272	M+H-H_2_O	213.091787; 185.096444; 142.078457; 154.078911; 157.101219
38	Inulicin	C_17_H_24_O_5_	75528891	326.1958085	261.257	0.587206957	M+NH_4_	85.028377; 326.196542; 183.101269; 267.122701; 165.091525
39	Bisabolangelone	C_15_H_20_O_3_	10399769	247.1340224	268.899	0.09080725	M−H	247.132609; 203.143286; 248.137682; 162.837881; 92.676764
41	Demethylnobiletin	C_20_H_20_O_8_	358832	389.1225257	284.678	1.21882624	M+H	389.119241; 359.077366; 374.098167; 341.063326; 356.089036
42	Alloimperatorin	C_16_H_14_O_4_	69502	271.0938387	292.379	0.594815307	M+H	271.094804; 105.382367; 92.132945; 192.297717; 259.592281
43	Polygodial	C_15_H_22_O_2_	5080908	235.169451	298.258	1.917771994	M+H	235.169945; 93.069512; 107.085554; 121.101703; 133.100577
44	Zedoarondiol	C_15_H_24_O_3_	14632997	253.1797	305.905	1.184805966	[M+H]+	93.069488; 81.069513; 121.10071; 79.054399; 107.085486
45	Gymnemagenin	C_30_H_50_O_6_	10051937	505.3525394	309.753	0.911444093	M−H	505.350093; 487.347686; 56.152842; 71.013656; 506.363847
46	Atractylenolide III	C_15_H_20_O_3_	155948	249.1486649	314.085	1.34514875	[M+H]+	249.14809; 119.08487; 92.661102; 147.079977; 252.132632
47	Bruceine A	C_26_H_34_O_11_	160006	539.2131534	321.513	0.284396213	M−H+H_2_O	539.205976; 113.024072; 85.029152; 75.008734; 71.013838
48	Marrubiin	C_20_H_28_O_4_	73401	331.1916448	321.6995	1.072398059	M−H	331.193862; 332.196479; 313.181246; 92.67761; 133.520065
49	Dihydroactinidiolide	C_11_H_16_O_2_	27209	181.1225766	326.329	3.183261855	M+H	181.122452; 135.11727; 163.112022; 107.085367; 121.100795
50	Butyl isobutyl phthalate	C_16_H_22_O_4_	28813	301.1413047	341.735	1.011821714	M+Na	301.141862; 286.047221; 81.069483; 184.650306; 92.656035
51	Saikosaponin D	C_42_H_68_O_13_	107793	803.4522042	372.685	2.235120875	[M+Na]+	803.463922; 803.507124; 92.654344; 641.40604; 625.930823
52	Terminolic acid	C_30_H_48_O_6_	12314613	503.3382395	385.895	0.475782683	M−H	503.341111; 55.928854; 504.338726; 311.375763; 110.400566
53	Crocetin	C_20_H_24_O_4_	5281232	329.1722963	404.626	0.900207345	[M+H]+	329.172948; 149.692598; 256.954708; 336.743959; 171.70608
54	AKBA	C_32_H_48_O_5_	11168203	513.35481	418.236	0.370144244	M+H	513.35574; 57.039088; 92.658572; 495.343888; 514.360992
55	Quillaic acid	C_30_H_46_O_5_	101810	485.3277089	430.64	1.460587582	M−H	485.32381; 53.927556; 486.328987; 92.675918; 379.981476
56	(4aS,6aS,6bR,9R,10R,11R,12aR)-10,11-dihydroxy-9-(hydroxymethyl)-2,2,6a,6b,9,12a-hexamethyl-1,3,4,5,6,6a,7,8,8a,10,11,12,13,14b-tetradecahydropicene-4a-carboxylic acid	C_30_H_48_O_5_	23757202	489.3567674	436.014	0.475344562	[M+H]+	205.160139; 191.144531; 453.341398; 203.17906; 489.324348
57	Pygenic acid B	C_30_H_48_O_5_	12308659	487.3421468	437.467	1.750717748	[M−H]−	487.347592; 488.34909; 54.15171; 111.009208; 92.68015
58	Apigenin	C_15_H_10_O_5_	5280443	269.0452442	455.382	2.809282436	[M−H]−	269.044494; 225.055691; 62.963937; 270.047442; 241.050221
59	Cimigenol	C_30_H_48_O_5_	16020000	471.347276	472.593	0.585572418	M+H-H_2_O	471.350356; 453.333917; 185.131764; 435.325276; 293.227612
60	Pomolic acid	C_30_H_48_O_4_	382831	471.3484584	482.522	0.972499137	M−H	471.346489; 52.374449; 472.34771; 133.289188; 134.079265
61	Corosolic acid	C_30_H_48_O_4_	15917996	473.3630846	515.5	0.178787465	[M+H]+	455.35577; 437.344813; 295.242818; 121.10064; 173.131926
62	Oleanolic acid 28-O-β-D-glucopyranoside	C_36_H_58_O_8_	14189384	663.4124935	536.728	0.743809988	M+HCOO	455.35524; 663.411329; 617.40069; 50.596811; 97.568097
63	20-Deoxyingenol	C_20_H_28_O_4_	11290503	315.1948027	551.025	2.546744331	M+H-H_2_O	315.191006; 133.100671; 125.676823; 189.090937; 51.139422
64	Methyl[8]-Shogaol	C_20_H_30_O_3_	91721121	319.2248125	569.836	0.587449289	[M+H]+	319.225535; 301.211427; 109.854958; 227.756837; 127.093095
65	11-Keto-beta-boswellic acid	C_30_H_46_O_4_	9847548	469.3315601	601.329	0.93730445	M−H	469.333839; 281.241547; 154.541827; 357.801014; 92.680998
66	9-hydroxy-1,4a-dimethyl-7-propan-2-yl-2,3,4,9,10,10a-hexahydrophenanthrene-1-carboxylic acid	C_20_H_28_O_3_	10448477	339.1902539	607.388	0.74860204	[M+Na]+	339.289571; 69.069705; 55.054574; 95.085925; 83.085622
67	Glaucocalyxin A	C_20_H_28_O_4_	10471963	350.2298112	663.461	0.539028221	M+NH_4_	210.111133; 350.229669; 192.100537; 82.760478; 113.752814
68	Enoxolone	C_30_H_46_O_4_	10114	469.3317111	665.256	0.615556275	[M−H]−	469.334369; 52.150157; 470.338904; 412.977183; 285.937884
69	Ursolic acid	C_30_H_48_O_3_	64945	439.3566644	718.354	0.763858235	[M+H]+	191.179464; 203.179014; 439.35968; 95.085887; 189.164795
70	Betulinic acid	C_30_H_48_O_3_	64971	455.3524115	720.026	1.292391888	[M−H]−	455.355122; 456.352381; 50.597151; 444.575521; 203.241857
71	Alpha-Boswellic acid	C_30_H_48_O_3_	637234	457.369799	754.031	3.933259197	[M+H]+	457.352924; 439.35553; 421.35036; 193.157987; 121.10064
72	20(S)-Protopanaxadiol	C_30_H_52_O_3_	11213350	483.3809242	760.816	0.156811831	[M+Na]+	483.387525; 53.708146; 240.276588; 217.502137; 480.633583
73	Stigmasterol	C_29_H_48_O	5280794	395.3678549	764.692	2.162277811	[M+H]+	395.368602; 147.117219; 81.069484; 145.102194; 159.116863
74	Eclalbasaponin I	C_42_H_68_O_14_	10079039	819.4495614	764.982	0.685051695	M+Na	819.456032; 819.50053; 91.050532; 365.109116; 203.052701
75	Micheliolide	C_15_H_20_O_3_	442279	266.1725499	769.589	1.691129387	M+NH_4_	266.172107; 92.65772; 129.988652; 147.429102; 141.661701

The table lists the compounds identified in LNE based on their retention time, exact mass, molecular formula, and fragmentation pattern. Ion mode indicates whether detection occurred in the positive ([M+H]+, [M+NH4]+, etc.) or negative ([M−H]−, etc.) electrospray ionization mode. Fragment ions represent the top five fragment *m*/*z* values detected in MS/MS analysis.

**Table 2:** Calibration curve data for the HPLC quantification of the five key flavonoids.

Compound	Calibration Curves	R^2^	Linear Ranges (mg/mL)
Ferulic acid	y = 1980x + 3.7665	0.999	0.005–0.2
Luteolin	y = 2542.8x + 3.0522	0.999	0.005–0.2
Latifolin	y = 1360.9x + 2.867	0.999	0.005–0.2
Apigenin	y = 1157.4x + 2.8478	0.999	0.001–0.4
Acacetin	y = 3451.6x + 3.184	0.999	0.001–0.2

**Table 3:** Contents of the five key flavonoids in LNE determined by HPLC.

No.	Compound Name	Retention Time (min)	Content (mg/g Extract)
1	Ferulic acid	16.221	6.25 ± 0.34
2	Luteolin	31.349	4.81 ± 0.46
3	Latifolin	32.571	1.01 ± 0.16
4	Apigenin	36.491	5.66 ± 0.35
5	Acacetin	46.549	3.12 ± 0.13

## Data Availability

Data will be made available on request.

## Supplementary Materials

The following supporting information can be downloaded at: https://www.mdpi.com/article/10.3390/cimb47070546/s1.

## Abbreviations

The following abbreviations are used in this manuscript:

LNE	Ethanolic extract of *Lobelia nummularia*
HPLC-QTOF-MS	High-performance liquid chromatography coupled with Quadrupole Time-of-flight mass spectrometry
TCM	Traditional Chinese Medicine
TNBC	Triple-negative breast cancer
ROS	Reactive oxygen species
MMP	Mitochondrial membrane potential
TFC	Total flavonoid content
RE	Rutin equivalent
TPC	Total phenolic content
GAE	Gallic acid equivalent
DPPH	2,2-Diphenyl-1-picrylhydrazyl
ABTS	2,2′-Azino-bis(3-ethylbenzothiazoline-6-sulfonic acid)
DMEM	Dulbecco’s modified eagle medium
DMSO	Dimethyl sulfoxide
CCK-8	Cell Counting Kit-8
PBS	Phosphate-buffered saline
JC-1	5,5′,6,6′-Tetrachloro-1,1′,3,3′-tetraethylbenzimidazolylcarbocyanine iodide
H_2_DCF-DA	2′,7′-Dichlorodihydrofluorescein diacetate
NAC	N-acetylcysteine
PPI	Protein–protein interaction
STRING	Search Tool for the Retrieval of Interacting Genes/Proteins
GO	Gene Ontology
KEGG	Kyoto Encyclopedia of Genes and Genomes
SDS-PAGE	Sodium dodecyl sulfate–polyacrylamide gel electrophoresis
