# Supplementary material for: Molecular Mechanisms of Lobelia nummularia Extract in Breast Cancer: Targeting EGFR/TP53 and PI3K-AKT-mTOR Signaling via ROS-Mediated Apoptosis
Source: Curr Issues Mol Biol. 2025 Jul 14;47(7):546. doi: 10.3390/cimb47070546 (PMC12293554; doi:10.3390/cimb47070546)
Supplement: Supplementary file 1 [file cimb-47-00546-s001.zip › Figure S1.pdf]

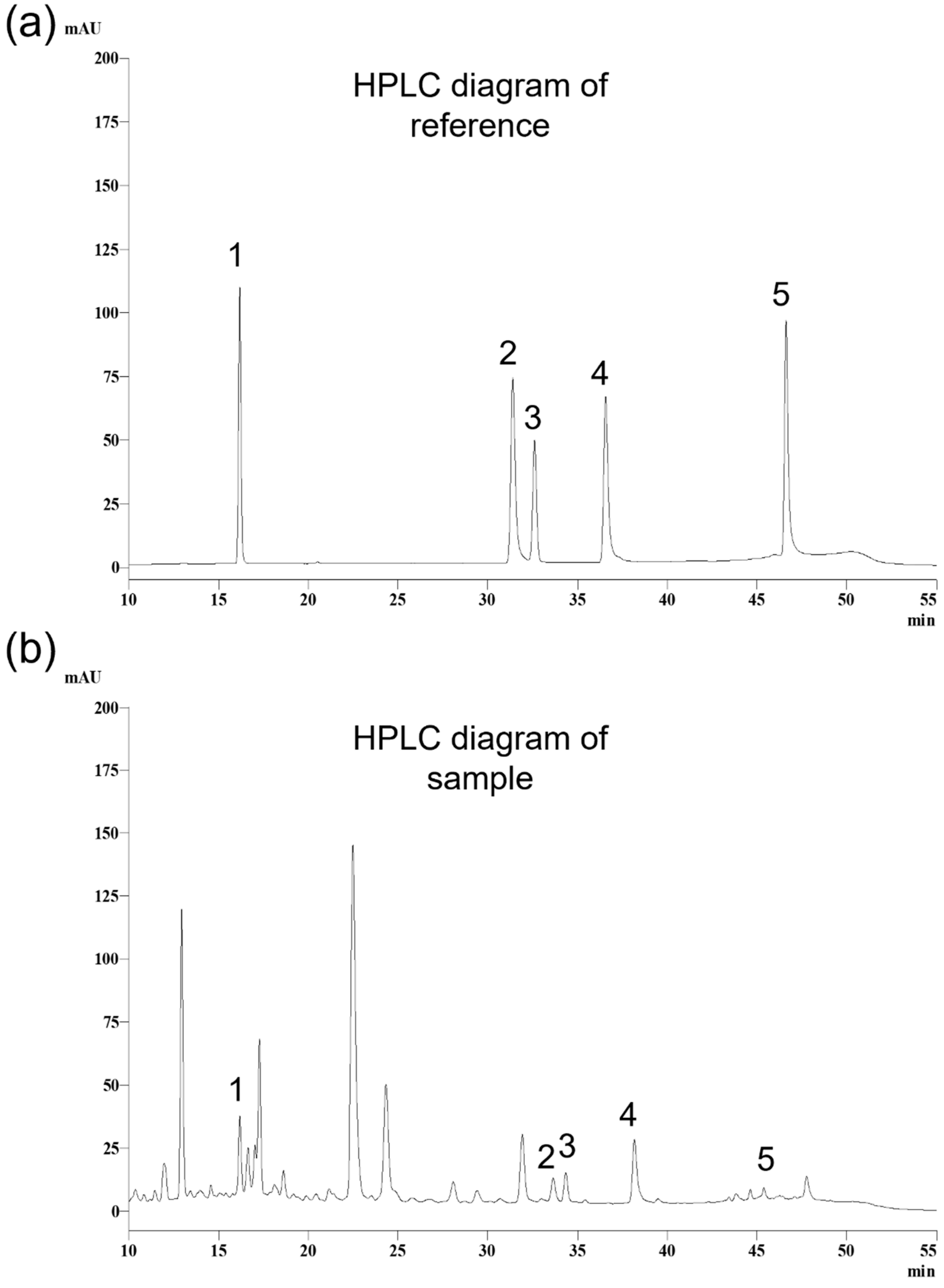

Figure S1. Representative HPLC chromatograms for the quantification of key flavonoids. (a) HPLC chromatogram of a mixed standard solution containing the five reference compounds. The peaks are identified as: 1. Ferulic acid, 2. Luteolin, 3. Latifolin, 4. Apigenin, and 5. Acacetin. (b) HPLC chromatogram of the *Lobelia nummularia* ethanolic extract (LNE). Detection was performed at a wavelength of 276 nm.
