# Supplementary figures and images for: Molecular Mechanisms of Lobelia nummularia Extract in Breast Cancer: Targeting EGFR/TP53 and PI3K-AKT-mTOR Signaling via ROS-Mediated Apoptosis
Source: Curr Issues Mol Biol. 2025 Jul 14;47(7):546. doi: 10.3390/cimb47070546 (PMC12293554; doi:10.3390/cimb47070546)

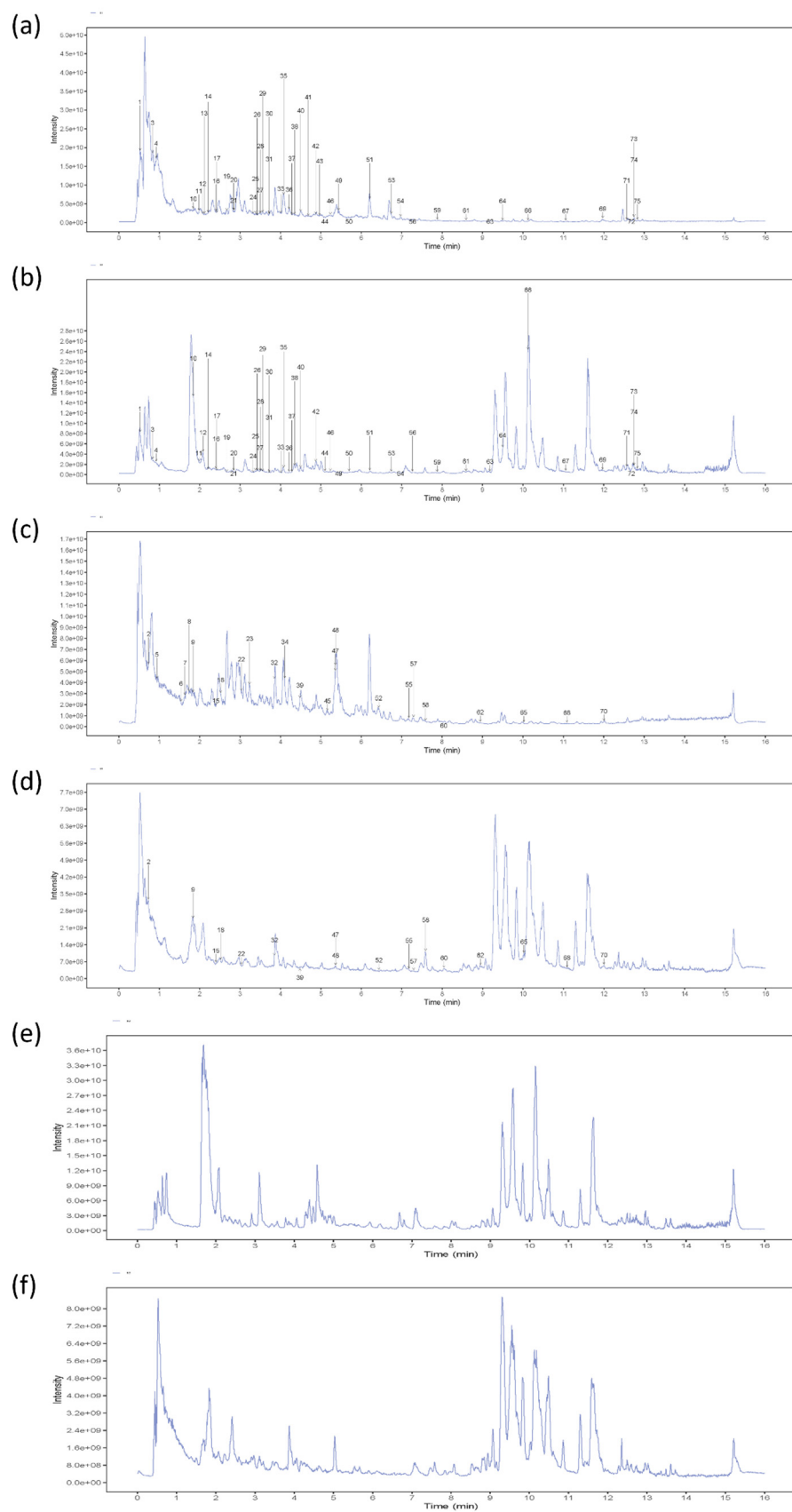

Supplement: Supplementary file 1 [file cimb-47-00546-s001.zip › Figure S2.pdf]
